# Development and validation of the Sensitivity to Intrusiveness Questionnaire

**DOI:** 10.3389/fpsyt.2026.1599703

**Published:** 2026-02-04

**Authors:** Aviv Akerman-Nathan, Jonathan D. Huppert, Eyal Kalanthroff

**Affiliations:** 1 Department of Psychology, Hebrew University of Jerusalem, Jerusalem, Israel; 2 Columbia University Irving Medical Center, Columbia University, New York, NY, United States

**Keywords:** cognitive consciousness, intrusive thoughts, intrusiveness, psychometrics, transdiagnostic questionnaire

## Abstract

**Introduction:**

Intrusive thoughts are involuntary mental experiences associated with various psychopathologies and can be disruptive. Although most research on intrusive thoughts focuses on negative content, intrusive thoughts are not defined solely by what they contain. Recent findings suggest that the distress associated with intrusive thoughts is driven more by individual characteristics than by the content of the thoughts themselves. To capture such content-independent individual differences, the present research focuses on the development and validation of the Sensitivity to Intrusiveness Questionnaire (STIQ).

**Methods:**

Across four studies conducted in different countries (*N* = 615), the psychometric properties of the STIQ were examined. Analyses assessed factor structure, internal consistency, test–retest reliability, and associations with personality traits and clinical groups.

**Results:**

Results supported a stable three-factor structure of the STIQ, reflecting negative experience of thoughts, awareness and monitoring of thoughts, and perceived lack of control over thoughts. The questionnaire demonstrated high internal consistency and test–retest reliability and showed meaningful variance across personality traits and clinical groups, independent of intrusive thought content.

**Discussion:**

The findings highlight the utility of the STIQ in capturing a transdiagnostic, content-independent sensitivity to intrusiveness. The STIQ provides a novel tool for examining individual differences and the mechanisms underlying distress associated with intrusive cognitions, contributing to a more comprehensive understanding of intrusiveness in psychological phenomena.

Intrusive thoughts are involuntary and repetitive mental intrusions that interfere with adaptive thinking, behavior, emotions, attention, and task performance (1). While these intrusive thoughts are commonly observed in psychopathological disorders, such as obsessive–compulsive disorder (OCD), post-traumatic stress disorder (PTSD), and depression (2–4), they also manifest in non-clinical populations (5–7). Interestingly, although these intrusive thoughts elicit significant distress, their content is not invariably negative (8, 9). Furthermore, research indicates that the content of intrusive thoughts does not necessarily differ between clinical and non-clinical populations (5, 10). These findings prompt the question of what accounts for the variance in how these intrusive thoughts are experienced, with some individuals finding them bothersome but manageable, others distressing and harmful, and some unbearable. Importantly, despite the prevalence and variety of intrusive thoughts throughout clinical and non-clinical groups, previous research on intrusive thoughts has predominantly focused on traumatic and other negative content thoughts due to their likelihood of becoming distressing. This line of research focused on the content of the thought as a main cause for the experience of intrusiveness. However, as we demonstrate below, the content of the thoughts alone cannot fully explain why some individuals perceive thoughts as aversive while others do not (9, 11). Thus, exploring the potential underlying mechanism causing the distress arising from intrusive cognitions, beyond content alone, might lead to a better understanding of these intrusions. The current paper describes the development of a questionnaire designed to quantify the extent of the intrusiveness experienced by an individual, thereby providing a mean to gauge individual differences in the sensitivity to such intrusions.

To gain deeper insight into the factors contributing to the aversiveness of intrusive thoughts among certain individuals, it is valuable to investigate the experiences of different involuntary and unwanted cognitions, such as repetitive thoughts, mind wandering, tinnitus, involuntary musical imagery (INMI, often referred to as ‘earworms’), and obsessions; each can cause significant distress, regardless of their content. These phenomena share common characteristics, including being challenging to control, occurring unintentionally, and capable of interrupting ongoing task engagement (9, 12–17). Specifically, both mind wandering, a state of having spontaneous thoughts unrelated to the ongoing activity, and INMI, the experience of having music parts repeatedly and involuntarily play in one’s mind, can manifest with content that is neutral, negative, or even positive (18–20). Yet, for some individuals, these experiences can still be perceived as intrusive and distressing. Similarly, tinnitus, the subjective perception of chronic high-pitched noise, lacks discernible content (21) but is often experienced as intrusive and aversive (22, 23). Most importantly, even in OCD the content of the obsessions is not necessarily negative; obsessions can consist of neutral content such as songs, noises, numbers, and non-violent images (e.g., clouds, numbers; 24). These examples provide compelling evidence for intrusions that are not solely defined by their negative content but can still be experienced as aversive by some individuals. Consequently, investigating mind wandering, INMI, and tinnitus presents an opportunity to explore the experience of intrusiveness irrespective of specific content (25). Specifically, by examining the aversive nature of these diverse phenomena, we may uncover shared underlying mechanisms contributing to the overall experience of intrusiveness.

One crucial factor that may underlie individual variations in the aversiveness of experiencing intrusive thoughts is the subjective sense of control over these intrusions. When a thought is perceived as highly distressing or threatening, both clinical and non-clinical individuals tend to make efforts to stop or suppress it (26–28). This tendency to exert control is often observed in mind wandering, INMI, and chronic tinnitus (14, 22, 29). However, individuals vary in their sense of control over these intrusive thoughts, leading some individuals to exert greater efforts to regain control (9, 30). In turn, these unsuccessful attempts to suppress these thoughts will ultimately increase meta-awareness of their recurrence (14, 31–33), fostering a sense of failure in controlling the thoughts and subsequently amplifying their occurrence (34–36). In other words, a low sense of control might lead to increased attempts to control the thoughts, often leading to an even lower sense of control. Importantly, this process is driven by a diminished sense of control over thoughts, irrespective of its content. An exemplary instance of this phenomenon is observed in individuals with OCD, who frequently describe an exaggerated and vigilant urge to monitor their thoughts (37–39). This, in turn, contributes to fostering the development of these thoughts into intrusive thoughts and diminishes the perceived sense of control over them. Indeed, studies on OCD have suggested that intrusive thoughts may be experienced as distressing due to a reduced sense of agency and the perception that one’s thoughts are not under one’s control (37, 40). Importantly, these theories shift the focus from the content of the thoughts to the characteristics of the individual. The interplay of meta-awareness, constant monitoring, and a reduced sense of control or agency over thoughts, regardless of their specific content, contributes to heightened distress in the presence of intrusive thoughts. These factors display significant variability among individuals, implying the feasibility of constructing a unified measure encompassing the ‘sensitivity to intrusiveness,’ which could assess individual differences in the tendency to perceive intrusive thoughts as more or less aversive. Hence, the need to extract control over thoughts could be one axis through which we assess the sensitivity to intrusiveness, independent of thought content.

Based on the notion that sensitivity to intrusiveness is a transdiagnostic characteristic independent of specific content, the current study aimed to construct a comprehensive ‘Sensitivity to Intrusiveness’ Questionnaire (STIQ) encompassing various types of cognitive intrusions. Across four empirical studies, we detail the development process of the STIQ and examine its psychometric properties and application to diverse groups diagnosed with psychopathologies. The STIQ represents a significant advancement in the study of intrusive thoughts by shifting the focus from content-driven appraisals to a transdiagnostic, content-independent measure of sensitivity to intrusions. Unlike existing questionnaires that primarily assess the frequency, content, or negative interpretation (appraisal) of intrusive thoughts, such as Obsessive Beliefs Questionnaire (41), the Obsessive–Compulsive Inventory – Revised (42), the Thought Control Questionnaire (43), or even the White Bear Suppresion Inventory (44), the STIQ captures a broader individual trait—the general tendency to experience thoughts as intrusive, distressing, and difficult to control, regardless of their specific content. This distinction is crucial, as prior research has shown that intrusive thoughts occur across both clinical and non-clinical populations, yet it remains unclear why some individuals find them highly distressing while others do not. In Study 1 we developed the STIQ by conducting a factor analysis to investigate the shared mechanism of intrusiveness across different types of cognitive intrusions. In Studies 2 we examined the reliability of the STIQ, in Study 3 we examined the relationship between the STIQ and personality traits, and in Study 4 we examined the STIQ across psychopathological groups.

## Study 1: Derivation and item selection

### Method

#### Participants

Two hundred and ten participants took part in the study in return for small monetary compensation (~10 USD). Participants were recruited through a PROLIFIC online surveys system and had to be between 18–70 years old, native speakers of English, currently residing in the US or the UK, and have an approval rate of at least 80% from at least 100 previous studies. Three attention-check questions (e.g., “*Please click ‘a lot’ for this question*”) were included in the questionnaire pack to examine participants’ task engagement and attention. Fifteen participants failed to answer two or more of these questions and were excluded from further analysis (7.14% of the participants). Additionally, we established predetermined criteria to exclude participants who failed to complete the questionnaires within the 30-minute time limitation or finished all questionnaires in less than 5 minutes, as this indicates low task engagement. In Study 1, no participant was excluded due to these criteria. Thus, the final sample included 195 participants (89 males and 106 females; mean age = 36.27, *SD* = 12.25). The current sample size is consistent with previous studies (45).

#### Procedure

The study was carried out online via PROLIFIC survey system. After signing an informed consent form, participants were asked to complete 8 self-report questionnaires, administered in a counter-balanced order. The presentation of the questionnaires and data collection were carried out via Qualtrics^XM^ software. Instructions emphasized that participants should pay full attention to the questions presented and that phones or any other distractors should be turned off or put away.

#### Questionnaires

The Obsessive–Compulsive Inventory – Revised (OCI-R; 42) is an 18-item self-report measure that assesses OCD symptoms. Each item is scored on a 5-point Likert Scale. Items are divided into 6 subscales (washing, checking, ordering, obsessing, hoarding, and neutralizing). In our sample, Cronbach’s alpha for the total score was .92, and for each subscale ranged from.81 (checking) to .86 (obsessing), compatible with previous studies (42).

The Metacognition Questionnaire-30 (MCQ-30; 46) is a 30-item self-report measure, designed to assess beliefs about thinking, worry, and intrusive thoughts. Each item is scored on a 5-point Likert Scale. Items are divided into 5 subscales (positive beliefs about worry, negative beliefs about uncontrollability and danger of worry, cognitive confidence, need for control, and cognitive self-consciousness). In our sample, Cronbach’s alpha for the total score was .94, and for each subscale ranged from .81 (Need to control thoughts) to .88 (Positive beliefs about worry), compatible with previous studies (46).

Ruminative Responses Scale (RRS; 47; based on the 22-item questionnaire; 48) is a revised 10-item self-report measure that assesses the individual’s tendency to engage in ruminative thoughts or behavior. Each item is scored on a 4-point Likert Scale. In our sample, Cronbach’s alpha for the questionnaire was.96, compatible with previous studies (49).

International Tinnitus Inventory (ITI; 50) is an 8-item questionnaire measuring the degree of distress and impact of tinnitus on an individual. Each item is scored on a 5-point Likert Scale. In our sample, Cronbach’s alpha for the questionnaire was.86, compatible with previous studies (50).

Tinnitus Questionnaire 12 (TQ-12; 51) is a 12-item self-report measure that assesses the most commonly reported adverse effects of tinnitus. Each item is scored on a 5-point Likert Scale In our sample, Cronbach’s alpha for the questionnaire was .91, similar to previous studies (52).

Mind-Wandering Questionnaire (MWQ; 53) is a 5-item scale for rapidly assessing trait levels of mind-wandering. Each item is scored on a 5-point Likert Scale. In our sample, Cronbach’s alpha for the questionnaire was .89, similar to previous studies.

Penn-State Worry Questionnaire (PSWQ; 54) is a 16-item self-report scale designed to measure the trait of worry in adults. The scale measures excessiveness, generality, and uncontrollable dimensions of worry. Each item is scored on a 5-point Likert Scale. Cronbach’s alpha for the questionnaire was .83, similar to previous studies (55).

### Results

#### Item selection for the STIQ

The process of selecting the items for the STIQ was conducted as follows:

(a) In the initial phase, we compiled a comprehensive set of relevant questionnaires (described above) based on a predetermined list developed by the research team. These questionnaires encompassed a total of 103 items.

(b) Subsequently, guided by theoretical considerations regarding relevant and aligned with our focus, we excluded items perceived as less pertinent to our goal. This process yielded a pool of 43 potential items related to intrusiveness (as detailed in **Supplementary Material 1**).

(c) As mentioned earlier, eleven items originally designed to assess cognition-specific aspects were adapted to gauge general intrusive thoughts and self-references. For example, an item from the Tinnitus Questionnaire (TQ-12; 51) originally reading, “*I am aware of the noises from the moment I get up to the moment I sleep*” was modified to a state item as follows: “*I am aware of my thoughts from the moment I get up to the moment I sleep*”. The complete list of modified items can be found in **Supplementary Material 2**. *All final STIQ items refer to thoughts in general and do not include any tinnitus- or modality-specific content.*

(d) An initial exploratory factor analysis (EFA) was conducted on the 43 selected items. Factor extraction was conducted using parallel analyses (56, 57), using 5,000 iterations and a mean Eigenvalue criterion (only factors with Eigenvalue exceeding the mean Eigenvalue from random, non-correlated data are retained). The parallel analysis revealed four latent factors. Based on our hypothesis that the potential factors of the sensitivity to intrusiveness are related, the EFA was carried out with a principal components’ method, with a fixed four factors solution using Direct Oblimin rotation (58), and items displaying a loading below 0.5 with the total score were excluded. As a result, a final set of 30 items remained.

(e) A second EFA was conducted to unveil potential subscales and ensure no unrelated subscales emerged. This second factor analysis on the remaining 30 items, identified four distinct and relevant subscales which explained a total of 67.08% of the variance. The first factor, which accounted for 43.352% of the total variance, consisted of items related to a negative experience of the thought (e.g., “*I am a victim of my thoughts*”). The second factor, which accounted for 11.75% of the total variance, consisted of five items all taken from the mind wandering questionnaire (53). The third factor, which accounted for 7.21% of the total variance, consisted of items related to monitoring of thoughts (e.g., “ *I monitor my thoughts*”). The fourth factor, which accounted for 4.76% of the total variance, consisted of items related to controlling thoughts or cognitions (e.g., “*I find it difficult to control my own thoughts*”). Since the second factor consisted only of the five mind wandering questionnaire items which failed to load on any of the other factors to a minimum of 0.5, they were thus excluded^1^. This process left us with 25 items. The rotated component matrix of the items loading before removing the five items can be found in **Supplementary Material 3** (in which the five removed items are marked).

(f) A third and conclusive EFA was performed on the remaining 25 items, with a fixed three factors solution using Direct Oblimin rotation (58), and items displaying a loading below 0.6 with the total score were excluded. As a result, a final set of 20 items remained. Accordingly, the following EFA accounted for a total of 68.99% of the variance. The first factor, which accounted for 50.08% of the total variance, comprised items associated with the negative experience of thoughts/cognitions (e.g., “*I find it harder to relax because of my thoughts*”, “*I am a victim of my thoughts*”), and was thus labeled “Negative experience of thoughts”. The second factor, which accounted for 11.23% of the total variance, comprised items associated with thought awareness and thought monitoring (“*I think a lot about my thoughts*”, “*I pay close attention to how my mind works*”) and was thus labeled “Awareness and monitoring the thoughts” (see **Table 1**). The third factor, which accounted for 7.68% of the total variance, comprised items associated with thought control (e.g., “*I find It difficult to control my thoughts*”), and was thus labeled “Lack of control”. After removing items below 0.6 loading, the final analysis retained 20 items (**Table 1**).

**Table 1:** Final STIQ items factor loadings.

STIQ item	Factor loading		
	1	2	3
Factor 1: Negative experience of thought			
1. Over the past two weeks, how much have the thoughts affected your sleep?^1^	**0.838**		
2. Over the past two weeks, how much have the thoughts affected your peace of mind?^1^	**0.877**		
10. Overall, how much have the thoughts affected the things you can do?^1^	**0.889**		
11. I am aware of the thoughts from the moment I get up to the moment I sleep^2^	**0.607**	0.437	
3. I find it harder to relax because of the thoughts^2^	**0.811**		
15. My thoughts are often so bad that I cannot ignore them^2^	**0.652**		
19. I am a victim of my thoughts^2^	**0.543**		−0.365
20. The thoughts have affected my concentration^2^	**0.639**		−0.327
14. Thoughts cause stress^2^	**0.745**		
Factor 2: Awareness and monitoring the thoughts			
7. I think a lot about my thoughts^3^		**0.553**	
12. I monitor my thoughts^3^		**0.823**	
13. I am constantly aware of my thinking^3^		**0.718**	
17.I pay close attention to how my mind works^3^		**0.852**	
4. I constantly examine my thoughts^3^		**0.718**	
Factor 3: Lack of control			
5. I find it difficult to control my own thoughts^4^			**−0.628**
6. I am upset by unpleasant thoughts that come into my mind against my will^4^	0.421		**−0.43**
8. Worrying persists even when trying to stop^3^			**−0.821**
9. I cannot ignore my worrying thoughts^3^			**−0.798**
18. I cannot stop worrying^3^			**−0.782**
16. Once I start worrying, I can't stop^5^			**−0.846**

The numbers represent the item loadings on the relevant factor, after the Exploratory Factor Analysis process. Coefficients below the value of 0.3 were suppressed and not printed in the table. The bold numbers point to the factor to which the item was eventually loaded.

Superscript reference:

^1^ Item drawn from International Tinnitus Inventory (ITI).

^2^ Item drawn from Tinnitus Questionnaire.

^3^ Item drawn from Metacognitive Questionnaire 30 (MCQ-30).

^4^ Item drawn from Obsessive–Compulsive Questionnaire Revised (OCI-R).

^5^ Item drawn from Penn State Worry Questionnaire (PSWQ).

#### Weighting adjustments

The factor analysis of the STIQ revealed a three-factor model, however, the number of items within each factor differs, necessitating the application of weighted scores to ensure that each factor contributes equally to the overall STIQ score. The largest factor, *“negative experience of thoughts*, consists of 9 items. The *“awareness and monitoring of thoughts”* factor is comprised of only 5 items. To balance its influence relative to the *Negative Experience of Thoughts*, a weight of 1.8 was applied to each item within this factor. Similarly, the *“lack of control”* factor, consisting of 6 items, thus, a weight of 1.5 was assigned to each item in this factor.

#### Reliability

Final reliability is reported by examining the internal consistency of each factor. Since Cronbach’s alpha is a function of the scale’s length (59), alphas were computed along with mean inter-item correlations and with corrected item–total correlations. As can be expected following the series of EFAs, the full scale of 20 items yielded excellent internal consistency as the general alpha = .946. When examining the subscales, all three subscales exhibited good to excellent levels of internal consistency: Negative experience of the thoughts, alpha = .93 (mean interclass correlation .61; item–total correlations .48–.73), Lack of control, alpha = .86 (mean interclass correlation .56; item–total correlations .47–.63), and Awareness and monitoring of thoughts, alpha = .89 (mean interclass correlation .59; item–total correlations .52–.88). Pearson correlations were computed to assess the relationships between the subscales. As shown in **Table 2**, Negative experience of thoughts was positively correlated with Awareness and monitoring of thoughts, (*r* = .73, *p* <.001), and Lack of Control, (*r* = .51, *p* <.001). Lack of control and Awareness and monitoring of thoughts demonstrated a positive correlation with each other, (*r* = .52, *p* <.001; **Table 2**). The specific contribution of each item to the general alpha is demonstrated in Supplementary.

**Table 2:** Descriptive statistics and correlations of STIQ scales.

Monitoring of thoughts	Lack of control	Negative experience of the thought	*SD*	*M*	*n*	Variable
.77**	.89**	.89**	19.46	55.68	203	STIQ (full questionnaire)
Subscales:						
–	–	–	8.58	22.22	203	Negative experience of the thought
–	–	0.51**	6.65	18.41	203	Lack of control
–	0.52**	0.73**	7.52	15.05	203	Awareness and monitoring the thoughts

Descriptive statistics of the Sensitivity to Intrusiveness Questionnaire (STIQ). *M*, mean; SD, standard deviation. ^**^p < .01.

## Study 2: Psychometrics validation of the STIQ

Study 2 was designed to investigate the psychometric properties of the STIQ further. Specifically, the main objective of this study was to examine the test–retest reliability of the STIQ, thereby evaluating the stability of the sensitivity to intrusiveness construct over time. To achieve this goal, participants completed the questionnaire twice, with a 2-week interval between administrations.

### Method

#### Participants

One hundred and twenty participants, who did not participate in Study 1, took part in the study for small monetary compensation (~8 USD). Participants’ recruitment and exclusion inclusion criteria were identical to Study 1. In total, 102 participants completed both versions of the STIQ. The same exclusion criteria for low task engagement as in Study 1, were applied here and four participants (3.9%) were excluded due to the time limitation criterion (no participants were excluded due to failing the attention check questions). The final sample included 98 participants (44 males and 54 females; mean age = 37.42, *SD* = 12.12). A power analysis using G*Power 3.1 (60) indicated that the current sample allowed for examination of the Pearson correlation between the two time points at a power > 95% to test large effects size (*F* = .40) with one tail and a Type I error (α <.05).

#### Procedure

All analyses were conducted using the statistical software SPSS (IBM Corp.; version 23). The study was carried out online via PROLIFIC survey system. While registering for the study, participants were informed that they would be contacted for a following study within exactly two weeks; they were not informed of the content of the following study. After signing an informed consent form, participants were asked to complete the STIQ (see **Supplementary Material**). The presentation of the questionnaires and data collection were carried online out via Qualtrics^XM^ software. Instructions emphasized that participants should pay full attention to the questions presented and that phones or any other distractors should be turned off or put away. The instructions of the STIQ were as follows: “*Please read each statement and choose the answer that indicates how much the statement applied to you over the past week (Did not apply to me at all/Applied to me to some degree, or some of the time/Applied to me to a considerable degree, or a good part of time/Applied to me very much, or most of the time). There are no right or wrong answers. Do not* sp*end too much time on any statement*”. Precisely two weeks (within a 4-hour window) after the first administration of the STIQ, participants were contacted through PROLIFIC and were asked to complete the STIQ again.

### Results

First, general alpha Cronbach for the entire questionnaire was calculated for the two time points, revealing excellent internal consistency for the first (.97) and second (.87) administration of the questionnaire. To assess the test–retest reliability of the STIQ, a Pearson’s correlation coefficient was calculated between the total STIQ score of the two time-points. The results revealed a significant and strong positive correlation (*r* = .70), indicating good test–retest reliability for the STIQ total score. All 3 factors displayed significant test–retest correlations (**Table 3**). When examining the individual items, all 20 STIQ items showed significant test–retest correlations, ranging from 0.56 (item 5, **Table 1**) to 0.73 (item 2, **Table 1**). The average measure of the Intra-Class Correlation (ICC) using a two-way mixed model was.91 with a 95% confidence interval ranging from.86 to.94 (*F* (99,99) = 11.05, *p* <.001). These findings indicate that participants’ tendency to experience intrusiveness remained stable over time, suggesting that this construct is a rather consistent trait within individuals. Finally, to examine whether the factor structure of the STIQ replicates the structure found in Study 1, a CFA was conducted using AMOS (version 29; 61) on the first administration of the questionnaire (Time 1). A good model fit was defined according to the following criteria: root mean square of approximation (RMSEA) ≤.06; comparative fit index (CFI) ≥.95; Tucker-Lewis index (TLI) ≥.95 (62). Our result yielded a significant Satorra-Bentler Chi-square (χ2 (162) = 230.98, p <.001), RMSEA of.06 (90% CI [.042,.078]), CFI of .97, and TLI of.96, indicating a good model fit.

**Table 3:** Pearson correlations of test–retest of STIQ scales.

Pearson r	SD-2^nd^	Mean-2^nd^	SD-1^st^	Mean-1^st^	Variable
.69**	13.80	46.61	27.29	63.20	STIQ
.64**	4.65	13.94	9.03	21.02	Negative experience of the thought
.66**	5.21	16.32	9.91	20.25	Awareness and monitoring the thoughts
.56**	5.01	16.33	9.33	21.93	Lack of control

Descriptive statistics of the first (baseline) and second (two weeks later) administration of the Sensitivity to Intrusiveness Questionnaire (STIQ) and its three subscales. Pearson correlation was carried out to investigate the test–retest reliability of the STIQ and its subscales. SD, standard deviation. ^**^p < .01. Interestingly, STIQ scores were lower in the second administration compared to the first one (t(97)=9.02, p<.01).

## Study 3: Sensitivity to intrusiveness and personality traits

After establishing the structure and basic psychometrics of the STIQ in Study 1 and Study 2, in Study 3 we aimed to examine whether specific traits are related to intrusiveness. As was mentioned in the Introduction, the STIQ aims to evaluate the multifaceted nature of sensitivity to intrusiveness and its impact on individuals’ subjective experiences. Central to this inquiry is the notion that not all individuals react uniformly to intrusive cognitions. While some individuals may exhibit a heightened sensitivity, experiencing intrusions with greater intensity and frequency, others may demonstrate a comparatively lower susceptibility, greater ability to ignore, or even indifference to such thoughts. One potential factor likely to affect this individual variability in the sensitivity to intrusiveness is personality traits. Thus, Study 3 aimed to investigate the correlations between the STIQ and different personality traits.

**Table 4:** Results of Study 3.

Lack of control	Monitoring of thoughts	Negative experience of thoughts	STIQ total	Big 5 scales
−0.14	−0.06	−0.08	−0.11	*Extraversion*
.70**	.67**	.34**	0.64**	*Neuroticism*
−0.09	−0.06	−0.07	−0.08	*Agreeableness*
−0.12	−0.09	−0.04	0.03	*Consciousness*
.27**	.30**	.33**	0.32	*Openness to experience*

Pearson correlation of the Sensitivity to Intrusiveness Questionnaire (STIQ) and its three subscales, with the Big 5 subscales. ^**^p < .01.

Individual personality traits play a pivotal role in shaping the way individuals process and react to subjective experiences. The Big Five model (63, 64), the most widely accepted taxonomy of personality, encapsulates five distinct dimensions: extraversion, agreeableness, conscientiousness, emotional stability (or neuroticism), and openness to experience. These dimensions collectively capture the most salient and enduring aspects of an individual’s personality and have been associated with a plethora of cognitive, emotional, and behavioral outcomes (65–67). Moreover, prior research has indicated that some personality traits are intertwined with various forms of psychopathology (68). For example, while emotional stability, which is the inverse of neuroticism, has been consistently found to be significantly associated with vulnerability to psychopathology (69), extraversion and agreeableness have been identified as contributors to positive mental health due to everyday satisfaction with social surroundings, romantic life, and acquaintances (70–72). If the STIQ is a measure of the subjective experience of intrusive cognition, it is expected to hold some relation to these overarching personality dimensions. Specifically, individuals scoring high on neuroticism, which is characterized by tendencies toward anxiety, worry, and emotional instability, are expected to possess heightened sensitivity to intrusive cognitions. Conversely, those high in extraversion, which is characterized by a tendency to be assertive and social, and to experience positive affect and seek excitement, might tend to redirect or refocus when faced with negative intrusive thoughts, given their positive emotionality, which is expected to result in lessened sensitivity to intrusiveness.

Study 3 aimed to investigate the relationship of the STIQ with personality traits, by examining its correlation with the comprehensive Big 5 questionnaire. Since the STIQ is intended to gauge sensitivity to intrusiveness, a trait often elevated in various psychopathologies, we hypothesized that neuroticism would show a positive correlation with STIQ scores, while extraversion would exhibit a negative correlation. The exploration of the relationship between the sensitivity to intrusiveness and specific traits not only enhances our understanding of the STIQ’s utility but also contributes to the broader comprehension of how personality traits intersect with sensitivity to intrusiveness.

### Method

#### Participants

One hundred and seventy undergraduate students, who did not participate in any of the other studies in the paper, took part in the study for partial course credit. Participants were recruited through the University Experiment Registration system and had to be between 18–70 years old and native speakers of Hebrew. The same exclusion criteria for low task engagement, as in Studies 1 & 2, were applied here and 17 participants (10%) were excluded due to failing the attention check questions, and 2 participants (1.17%) were excluded due to the time limitation criterion. The final sample included 151 participants in total (15 males and 136 females; Mean age = 22.76, *SD* = 1.83). A power analysis using G*Power 3.1 (60) indicated that the current sample allowed for examination of linear multiple regression with 5 predictors at a power > 80% to test medium to large effects size (*F* = .40) with a Type I error (α <.05).

#### Procedure

After signing an informed consent form, participants were asked to complete the STIQ (see **Supplementary Material**) and the Big Five Inventory (BFI) self-report questionnaire, administered in a counter-balanced order. The presentation of the questionnaires and data collection were carried out online via Qualtrics^XM^ software. The instructions and administration were identical to those of Study 2.

For the current study, the STIQ was translated into Hebrew. The translation process was conducted as follows: (a) the items taken from other questionnaires were adopted from validated Hebrew versions of these questionnaires. (b) the minor edits on the items that were changed for the STIQ (see Study 1) were translated and back-translated to ensure accurate translation.

#### Questionnaires

The “Big Five” Inventory (BFI; 73) is a 44-item self-report measure of five personality factors: extraversion (8 items; e.g., “*Like to talk a lot*”), agreeableness (9 items; e.g., “*Helpful and not selfish in relation to others*”), openness to experiences (10 items; e.g., “*Original, invents new ideas*”), consciousness (9 items; e.g., “*Does a thorough job*”), and neuroticism (8 items; e.g., “*Can be stressed out*”). Participants are asked to rate the degree to which each statement describes them on a 5-point scale (ranging from 1 – strongly disagree to 5 – strongly agree). In our sample, Cronbach’s alpha ranged from .34 (consciousness) to .79 (neuroticism).

#### Statistical analysis

All analyses were conducted using the statistical software SPSS (IBM Corp.; version 23). To assess the relationship between the STIQ and the Big 5 subscales, Pearson correlations were conducted. To assess the contribution of each Big 5’s subscale in the explained variance of the STIQ, a multiple regression was conducted, with extraversion, agreeableness, conscientiousness, openness to experiences, and neuroticism as the predictors, with STIQ as the dependent variable.

### Results

First, general alpha Cronbach for the entire questionnaire was calculated, revealing excellent internal consistency (.949), similar to Study 2. Next, Pearson correlations between the five assembled Big 5 subscales and the STIQ were carried out^2^. In line with our *a priori* hypothesis, STIQ scores demonstrated a strong positive correlation with neuroticism (*M* = 24.77, *SD* = 5.26), *r* = .64, *p* <.001. Surprisingly, a moderate positive correlation was also observed between STIQ and openness to experiences (*M* = 29.58, *SD* = 3.64), *r* = .32, *p* <.001. All other correlations were non-significant [extraversion (*M* = 21.35, *SD* = 4.12) *r* = −.11, *p* = .19; agreeableness (*M* = 31.31, *SD* = 3.89) *r* = −.08, *p* = .31; consciousness (*M* = 29.91, *SD* = 3.29) *r* = .03, *p* = .74). A multiple regression analysis revealed that only neuroticism (*β* = .63, *t* = 10.62, *p* <.001) and openness to experience (*β* = .35, *t* = 4.29, *p* = >.001) were significant positive predictors of STIQ. In block-wise entering, neuroticism added 30% of the variance explained (*F ^change^* (1,134) = 74.38, *R*^2 change^ = .31, *p* <.001), and openness to experience added 12% of variance explained (*F ^change^* (1,135) = 18.42, *R*^2 change^ = .12, *p* <.001).

### Discussion

The present study aimed to investigate the relationship between STIQ and personality traits by examining its correlation with the Big 5 personality traits. The results of this study indicated that STIQ was positively and strongly correlated with two of the Big 5 scales: neuroticism and openness to experience. The neuroticism scale describes the overall emotional stability of an individual, and how they interpret events in the world. Individuals high on neuroticism are more likely to experience negative affect, anxiety, self‐consciousness, and depression (74). Thus, the correlation found in the current study suggests that individuals who score higher in neuroticism are likely to be more sensitive to intrusiveness, though the causal connection between the two cannot be determined. The fact that the STIQ was positively correlated with neuroticism is not surprising, since the STIQ is expected to be linked with symptoms related to psychopathologies such as negative emotions, obsession, rumination, anxiety, worry, and uncontrollability, all of which have been shown to be related to neuroticism (75–77). Thus, the strong positive correlation between neuroticism and the STIQ further supports the validity of the questionnaire as a measure of intrusive experiences. In addition, by exploring the association between the STIQ and the neuroticism scale of the Big 5 questionnaire, this study sought to shed light on the degree to which the STIQ can be considered a distinct construct beyond the existing concept of neuroticism. The neuroticism scale accounted for 30% of the variance in the STIQ, suggesting that while neuroticism is certainly a relevant aspect of sensitivity to intrusiveness, the STIQ is not fully accounted for by neuroticism and other factors influence the intrusiveness experience.

Contrary to our hypothesis, the results of the current study revealed that extraversion does not exhibit a negative correlation with the STIQ scores. Extraversion is not a monolithic, homogeneous trait; it represents a spectrum where individuals are variably placed (78). At the higher end of this spectrum, individuals displaying significant extraversion tend to show traits associated with overall well-being, adaptive coping mechanisms, and a heightened sense of life satisfaction (79, 80). In contrast, those with lower levels of extraversion, often categorized as introverts, are predisposed to depressive states, social anxiety (81, 82), and even disinhibition (68). Interestingly, several studies suggested that low extraversion has been linked to OCD, particularly when accompanied by high levels of neuroticism (83–86). Taken together with our results, this might imply that extraversion by itself cannot predict the individual variety in the sensitivity to intrusiveness. Future research should inquire into personality combinations that are more prevalent in sensitivity to intrusiveness.

A positive correlation was found between the openness to experience scale and STIQ scores. This correlation is surprising and yielded minimal significance in the multiple regression analysis and should thus be considered with caution. Openness to experience represents a broad personality trait that includes imagination, creativity, and a willingness to embrace novel experiences (78, 87). Previous studies demonstrated that openness to experience is related to a more efficiently functioning Default-Mode Network, which, among other things, is associated with mind-wandering, future thinking, and creative idea production (88). Thus, it is possible that individuals who score higher in openness to experience tend to focus their attention on their cognitions, and therefore, are more aware and disturbed by interruptions and repetitiveness of cognitions. Intensely focusing on the inner workings of one’s mind can potentially lead to (unsuccessful) efforts to control it, resulting in a more negative experience of these cognitions (26, 27). This heightened attention to internal mental processes may further amplify an individual’s sensitivity to intrusiveness. However, it is important to emphasize that these speculations regarding the underlying mechanism of this correlation should be considered with caution given the unexpected nature of this finding. Further investigation is warranted to better understand the underlying mechanisms and implications of this unexpected correlation. One limitation of this study is the unequal distribution of male and female participants, which reflects the composition of the student sample and restricts the generalizability of these findings across sexes.

To conclude, the results of Study 3 suggest that the STIQ is strongly associated with the specific personality traits of neuroticism, and much less, or not at all, with other personality traits. Given the literature review above and the nature of neuroticism and other personality traits, this finding supports the construct validity of the STIQ. Further research could investigate whether sensitivity to intrusiveness mediates the relationship between personality traits and psychopathology and whether interventions aimed at reducing sensitivity to intrusiveness could be effective in individuals with specific personality traits.

## Study 4: Psychopathological groups

Study 4 was designed to investigate the STIQ across different psychopathological groups. As discussed above, individuals vary in their subjective experience when confronted with intrusive thoughts entering their consciousness. This variability, as our hypothesis suggests, might be attributed to inherent cognitive structures, individual differences, and/or neurobiological factors. The findings from Study 1 revealed that the STIQ comprises various components, including metacognition and monitoring, control over cognitions, and negative experiences related to intrusive thoughts. These facets often correspond with characteristics typical of specific psychopathologies. Furthermore, the results of Study 3 demonstrated a positive correlation between sensitivity to intrusions and individuals scoring on the Big-5 neuroticism scale, a trait associated with psychopathological tendencies (69, 89). Taken together, these findings lead us to predict that the STIQ scores will be higher among individuals diagnosed with specific psychopathologies characterized by intrusive cognition, compared to individuals without any psychopathology or to individuals diagnosed with psychopathologies *not* characterized by intrusive cognitions.

Examining the sensitivity to intrusiveness profiles across diverse psychopathological subgroups is important in assessing the STIQ’s construct validity. The DSM-5 (90) underscores disparities between psychopathological subgroups in their cognitive experiences and characteristics, suggesting that certain groups might be more predisposed to experiencing intrusive cognitions and have heightened sensitivities to such intrusions. The diagnostic criteria for disorders such as OCD, PTSD, and major depression disorder (MDD) explicitly highlight the presence of intrusive and unwanted thoughts as core symptoms (90–92). Indeed, a vast amount of research emphasized the heightened prevalence of intrusive thoughts in these disorders (OCD: 3, 92, 93. PTSD: 2, 94. MDD: 4, 95, 96). Thus, these three disorders are the top candidates for assessing STIQ, as we predict higher scores in those disorders compared to healthy controls (HC).

In order to assess a diverse and comparable clinical group that is not necessarily characterized by sensitivity to intrusive thoughts, we selected attention-deficit/hyperactivity disorder (ADHD), which is typified by a predilection for involuntary, non-intrusive thoughts, rather than distressing intrusive cognitions (97; 98–101)^3^. Importantly, intrusive thoughts are not described as a core diagnostic feature of ADHD in the DSM-5 (90), in contrast to disorders such as OCD and MDD, in which intrusive and unwanted thoughts are central to the clinical presentation.

Thus, in the current study, we wish to examine whether the STIQ enables differentiation between individuals who are sensitive to intrusiveness, by comparing the STIQ score of HCs to that of individuals diagnosed with MDD, OCD, and ADHD. Specifically, we hypothesized that the STIQ scores of the HC and the ADHD groups would be lower than the scores of the MDD and OCD groups, with no significant difference between the HC and ADHD groups and between the MDD and OCD groups.

### Method

#### Participants

One-hundred seventy-eight participants took part in the study for small monetary payment (~8 USD). The sample size, recruitment, and diagnosis procedures are elaborated below for each group separately. The same inclusion/exclusion criteria as in Study 1, 2, and 3, were applied here. The full demographic and clinical characteristics of the sample is presented in **Table 5**. A power analysis using G*Power 3.1 (60) indicated that the current sample allowed for examination of a one-way analysis of variance (ANOVA) at a power > 80% to test medium effects size (*F* = .25) with a Type I error (α <.05).

**Table 5:** Results of Study 4.

OCD	MDD	ADHD	HC	Group
53	17	40	64	N
31 / 21	4 / 13	8 / 31	8 / 55	N male / female
30.32	24.75	24.80	23.38	Age Mean
99.94 (18.74)	91.47 (22.63)	78.20 (22.13)	67.39 (20.67)	STIQ Mean (SD)
32.711 (6.10)	31.00 (7.16)	27.20 (7.05)	22.70 (6.94)	*Negative experience of the thought Mean*
34.07 (7.25)	29.03 (8.61)	24.92 (8.27)	21.63 (7.87)	*Monitoring of thoughts Mean*
33.16 (7.05)	31.44 (8.17)	26.07 (8.32)	23.06 (7.67)	*Lack of control Mean*

Descriptive statistics of the Sensitivity to Intrusiveness Questionnaire (STIQ) and its three subscales, in the different clinical groups of Study 4. HC, healthy control; OCD, obsessive–compulsive disorder; MDD, major depression disorder; ADHD, attention-deficit/hyperactivity disorder; SD, standard deviation.

Healthy Controls (HC). Sixty-eight participants who did not participate in any of the other studies took part in the study for small monetary payment (~8 USD) or partial course credit. Participants were recruited through the University Experiment Registration System. At the beginning of the study, all participants were asked the following question: “*Were you ever clinically diagnosed with one of the following diagnoses: OCD/ADHD/eating disorder/PTSD/depression/any other psychological/psychiatric diagnosis?*”. Next, they were asked if they ever suspected having one of these or any other form of psychopathology, or if anyone close to them ever suggested they do. Only participants who self-reported having no diagnosis of any psychopathology and no suspicions of having one were included in the sample. Five participants (7.3%) were excluded due to failing attention checks, and no participants were excluded due to the time limitation criterion.

Attention-Deficit Hyperactivity Disorder (ADHD). Forty participants were recruited through an ongoing (unrelated) study on ADHD. The formal diagnosis of ADHD was conducted by the Institute for the Diagnosis of Learning Disabilities and Attention Deficits (ELAH) at the Israel National Institute for Testing and Evaluations (NITE; established by the associated heads of the universities in Israel) and all participants were recognized as having ADHD by their academic institution. The ELAH comprehensive diagnosis includes 2 questionnaires, a full psychiatric interview, and 20 computerized tests that assess cognitive functions, language (reading and writing), arithmetic thinking, attention, memory, perception, and general processing speed. The diagnosis is conducted over 3 meetings: Two meetings of approximately two hours each, for the questionnaires and tasks, and 1 meeting for the in-person interview (for more details on this diagnosis procedure see 103). Importantly, the above-mentioned ADHD diagnostic procedure is completely compatible with DSM–5 criteria. Participants in the current study were asked to present a diagnosis completed by the ALAH institute within 3 years prior to the study. Individuals with comorbid Autistic Spectrum Disorders, MDD, PTSD, or OCD were excluded. Participants were asked to complete the STIQ between 2–4 weeks after participating in the original experiment for which they were recruited. One participant (0.03%) was excluded due to failing the attention checks, and no participants were excluded due to the time limitation criteria.

Major Depressive Disorder (MDD). Seventeen participants were recruited through an ongoing (unrelated) study on MDD. Initial screening was conducted using an online survey with four items taken from the Beck Depression Inventory II (BDI-II; 104) as part of a larger survey. Participants with the highest scores were invited to the lab. The diagnosis was determined by PhD-level licensed clinical psychologists, specializing in MDD, using the Mini International Neuropsychiatric Interview (MINI; 105). Individuals had to have a primary diagnosis of MDD with the severity being at least moderate. Exclusion criteria included any current comorbid disorder and use of medication. Participants filled out the STIQ immediately after the diagnostic procedure and prior to participation in any additional study. None of the participants were excluded due to time limitation criteria or due to failing attention checks.

Obsessive–Compulsive Disorder (OCD). Fifty-three participants were recruited through an ongoing (unrelated) OCD treatment study, as part of the OCD research clinical at the University. Eligibility was determined by PhD-level licensed clinical psychologists, specializing in OCD, using the Structured Clinical Interview for DSM-5 (SCID-5) and the Yale–Brown Obsessive–Compulsive Scale (Y-BOCS). Individuals had to have a primary diagnosis of OCD (duration of ≥1 year), and a Y-BOCS score ≥ 16. Exclusion criteria included manic episode (current or past), psychosis (current or past), current prominent suicidal ideation, substance abuse or dependence in the past 6 months, current severe major depressive disorder, comorbid ADHD, PTSD, or any neurological disorder. Patients were not excluded if they had other comorbid disorders, as long as OCD symptoms were the most severe and impairing diagnoses. Patients on psychiatric medication were eligible if they were on a stable dose for at least six weeks prior to the study^4^. Participants filled out the STIQ (see **Supplementary Material**) immediately after the diagnostic procedure (prior to participating in any clinical intervention). One participant (1.9%) was excluded due to the time limitation criterion, and none were excluded due to failing the attention checks.

#### Procedure

The presentation of the questionnaires and data collection were carried out online via Qualtrics^XM^ software. Instructions and procedures were similar to Studies 1–3.

#### Statistical analysis

All analyses were conducted using the statistical software SPSS (IBM Corp.; version 23). To evaluate the relationship between the groups and the sensitivity to intrusiveness, a one-way analysis of variance (ANOVA) was carried out on STIQ scores, with group (OCD, MDD, ADHD, and HCs) as a between-subject factor. To examine our *a priori* hypotheses, planned comparisons were carried out using orthogonal interaction contrasts: (1) HC *vs*. all other clinical groups; (2) HC and ADHD *vs*. OCD and MDD; (3) OCD *vs*. MDD; (4) HC *vs*. ADHD. Follow-up interaction contrasts included: (5) MDD *vs*. ADHD; and (6) OCD *vs*. ADHD.

### Results

First, a general alpha Cronbach for the STIQ was calculated, revealing excellent internal consistency in this sample (alpha = .958). The one-way ANOVA revealed a significant main effect for group (*F*(3,171) = 25.374, *p* < 0.001; see **Table 5**)^5^. The planned interaction contrasts revealed that: (1) The STIQ scores of the HC group were significantly lower than that of the clinical groups (*t*(168) = 6.60, *p* <.001). (2) The STIQ scores of the HC and ADHD were significantly lower than that of the MDD and OCD groups (*t*(168) = 6.42, *p* <.001). (3) Although the STIQ scores of the OCD group were slightly higher than those of the MDD group, this difference was not significant, (*t*(168) = 1.46, *p* = .14, *d* = −0.338). (4) Counter to our prediction, the STIQ scores of the HC group were significantly lower than those of the ADHD group (*t*(168)= 2.57, *p* = .01, *d* = .471), based on an equivalence test (106) with an equivalence range of *d* = −0.5 to 0.5, we conclude the absence of an effect we deemed meaningful. Follow-up analyses revealed that: (5) The STIQ scores of the ADHD group were significantly lower than that of the MDD group (*t*(54) = 2.05, *p* <.02, *d* = −.59) and of the OCD group (*t*(168) = 4.90, *p* <.001, *d* = −1.73)^6^.

### Discussion

The present study sought to elucidate the expression of the Sensitivity to Intrusiveness Questionnaire (STIQ) within diverse psychopathological groups. Consistent with our hypothesis, the STIQ scores of the MDD and OCD groups were significantly higher compared to those of the HC and ADHD groups, underscoring a heightened sensitivity to intrusions within those clinical groups (3, 95, 107). Consistent with the idea that sensitivity to intrusiveness is a transdiagnostic trait, there was no significant difference between the MDD and OCD groups. A small albeit significant difference in STIQ scores was observed between the ADHD and HC groups. This finding indicates that in contrast to our hypothesis, some individuals with ADHD are more sensitive to intrusions (108). However, the ADHD group scores were lower compared to the other clinical groups supporting the notion that individuals with ADHD do not necessarily experience intrusive thoughts as distressing as individuals with MDD and OCD do.

The findings of Study 4 have important implications: first, they suggest that the STIQ is sensitive to intrusiveness associated with certain psychopathologies. Our results indicate that while certain clinical groups do experience intrusiveness, the expression of their experience and the distress from it may differ. Secondly, these results imply that the cognitive intrusions characteristic of ADHD may be qualitatively different from the intrusive thoughts known in other clinical conditions and may cause less awareness and less distress. In conclusion, the current study adds to the growing body of evidence that underscores the importance of examining psychopathological symptoms through the prism of sensitivity to intrusiveness. The STIQ can serve as a valuable tool in differentiating between groups with high and low sensitivity to intrusive thoughts, particularly within clinical populations characterized by distressing intrusions.

Although OCD and MDD both involve intrusive thoughts, these intrusions differ phenomenologically. In OCD, intrusive thoughts are typically experienced as ego-dystonic, unwanted, and often threat-related, whereas in MDD intrusive thoughts are more commonly ruminative, repetitive, and mood-congruent (3, 109, 110). Despite these phenomenological differences, both disorders are characterized by heightened distress in response to intrusive cognitions, which is consistent with the conceptualization of sensitivity to intrusiveness as a transdiagnostic dimension underlying diverse clinical presentations (111, 112).

PTSD represents another highly relevant clinical population for examining sensitivity to intrusiveness, given the centrality of intrusive re-experiencing symptoms such as intrusive memories and flashbacks (113, 114). However, due to practical and methodological constraints related to recruitment and diagnostic verification, the current study was limited to the included diagnostic groups. Future research should extend the examination of the STIQ to post-traumatic stress disorder and other clinical populations in which intrusive experiences are central, in order to further refine and validate the transdiagnostic scope of the measure. A limitation of Study 4 is the relatively small size of the MDD group compared to the other diagnostic groups, resulting in unequal group sizes; At the same time, the inclusion of participants with verified clinical diagnoses across multiple groups remains a notable strength, enhancing the ecological validity and clinical relevance of the findings.

## General discussion

The current research aimed to introduce the concept of sensitivity to intrusiveness as a transdiagnostic dimension with implications for both clinical and healthy individuals. Existing research tools for investigating intrusiveness, particularly without negative content, are limited (115). As discussed in the introduction, intrusiveness can be experienced with neutral, or even positive, through thoughts, pictures, songs, or inner sounds. The content-neutrality of these intrusions underscores the argument that the impact and subjective experience are determined not only by the content of the thought but also by the individual’s sensitivity to its intrusiveness. Despite the absence of a dedicated tool to measure such sensitivity to intrusiveness, items from different questionnaires made indirect and incomplete references to the intrusiveness experience. Thus, in a series of studies, we constructed and validated the Sensitivity to Intrusiveness Questionnaire (STIQ). We found that a three-factor model fit the questionnaire and its subscales in both English and Hebrew and across different samples, and that the total score and subscales had excellent internal consistency. We also demonstrated that the STIQ demonstrated high test–retest reliability, indicating good internal stability. The STIQ also showed strong correlation with neuroticism, but not with other personality factors. Finally, individuals diagnosed with OCD and MDD had higher STIQ scores compared to HCs and individuals with ADHD, suggesting that the disorders can be differentiated by the STIQ measure of intrusiveness. In this light, the STIQ offers a promising tool for quantifying and understanding the sensitivity to intrusiveness at the individual level.

Intrusive thoughts are a key characteristic of various psychopathologies. While the existing body of literature predominantly focuses on the intrusive nature of negative thoughts, as emphasized by Clark (3), Greenberg (91), and Janeck et al. (38), scientific evidence and clinical experience reveal that intrusive thoughts are not solely limited to negative content; they may also encompass neutral or even positive content. Thus, note that some of the items in the STIQ refer to the negative *experience* of the intrusion, and not to the negative content. Throughout the four studies, the current paper unveils a significant aspect of variability in individuals’ responses to intrusive thoughts. This variability is attributed to the conceptualization of ‘sensitivity to intrusiveness’ as a continuum underlying individual differences across the full spectrum of mental health. In other words, two individuals with identical intrusive thought content may exhibit profoundly different levels of distress, emphasizing the nuanced nature of this psychological phenomenon (25). Importantly, our study contributes novelty to the field by recognizing and measuring ‘sensitivity to intrusiveness’ as a transdiagnostic characteristic, independent of specific content or clinical diagnosis. The acknowledgment of this continuum sheds light on why diverse unintentional cognitive experiences, such as tinnitus, INMI, thoughts, and obsessions, share commonalities, as demonstrated in Study 1. The tinnitus-based items represent an involuntary, internally generated experience that varies in perceived intrusiveness, mirroring the way intrusive thoughts differ in how distressing or disruptive they are perceived to be. This underscores the presence of a shared mechanism among individuals who are more vulnerable to the negative impact of foreign intrusions entering consciousness, transcending the specific content of the intrusion and across different psychopathologies. This conclusion has specific implications for the Research Domain Criteria (RDoC) initiative. The RDoC initiative aims to deconstruct constellations of symptomatology into dimensions closer to neurobiology and to rethink how we classify mental illness in a dimensional, rather than categorical, manner (116). In a previous attempt to map OCD symptoms onto existing RDoC domains and constructs (117), it was clear that repetitive behaviors (compulsions) and anxiety can be mapped onto the existing RDoC matrix, while intrusive thoughts (obsessions) are more difficult to map onto the existing domains and constructs. The STIQ provides a novel tool to investigate intrusiveness as a new domain.

The results of the current investigation suggest that several factors contribute to the variance of sensitivity to intrusiveness. One salient factor identified is a heightened awareness of recurring and repetitive cognitions, also known as Cognitive Self-Consciousness (CSC). Elevated CSC appears to render negative and neutral-valence thoughts more salient, thereby increasing the likelihood of negative thought appraisals (38). Heightened CSC has also been shown in OCD (118, 119). The consequential focus and excessive monitoring of consciousness further amplify this awareness (34–36, 120). Such a hyper-aware state can escalate the distress linked to these intrusive thoughts (34, 121). This vicious cycle of awareness and over-monitoring might explain the positive relationship between the openness to experience trait and the STIQ, demonstrated in Study 3. Interestingly, this heightened CSC seems intricately intertwined with an individual’s sense of cognitive control. Studies point towards a strong association between CSC and the experience of loss of cognitive control (122), mirroring the diminished sense of agency over thoughts (37, 123). The aspect of (loss of) control is also evident in several clinical groups, such as OCD and MDD (30, 124), which were more sensitive to intrusiveness, as evident in Study 4. On the other hand, the lower STIQ score of the ADHD group demonstrated in Study 4, suggests that while ADHD might be characterized by difficulties in cognitive control, often manifesting as distractibility, mind wandering and/or impulsivity, they are not necessarily characterized by heightened CSC.

Another factor that was found to be linked to sensitivity to intrusiveness in this study, is the personality trait known as neuroticism. Neuroticism is one of the core personality dimensions in the Big 5 model and was defined as the tendency of an individual to experience negative emotional states such as anxiety, moodiness, worry, and envy (125). This trait is often linked to emotional instability with maladjustment or negative emotionality (78). When viewed in the context of sensitivity to intrusiveness, several facets of neuroticism provide an illuminating perspective. Individuals high in neuroticism often display heightened emotional reactivity and are aroused quickly when stimulated (126). Such increased emotional responses can make these individuals more susceptible to perceiving their thoughts as intrusive or distressing, even if the content of the thought is neutral or pleasant. Their heightened sensitivity to emotional shifts can amplify the impact of any aware thought, making it harder for them to dismiss or disengage from it. Second, neuroticism can predispose individuals to interpret ambiguous thoughts in a negative or threatening light (126). A neutral thought, when passed through the ‘filter’ of neuroticism, might be interpreted as more menacing or worrisome than it objectively is. Interestingly, evidence for appraisal of unwanted thoughts was also demonstrated as a key factor contributing to the development and persistence of intrusive thoughts and OCD symptoms (10, 11, 45, 127). Thus, the interplay between the neuroticism scale and the STIQ emphasizes that the tendency to experience negative emotions is more likely to cause the aversive experience of (even neutral) thoughts, as was also demonstrated by Akerman-Nathan et al. (25). It seems that the personal appraisal of the occurrence of the thought might be an important factor contributing to the recurrence of the thought, and thus, to the aversive experience.

In addition to the general STIQ scale, the current study identified three dimensions of sensitivity to intrusiveness: negative experience of thoughts, awareness and thought monitoring, and lack of control. Study 2 reinforces the validity of the STIQ’s three-factor model, elucidating substructures that facilitate a more detailed comprehension of the overarching construct of intrusiveness. Although many STIQ items reflect negatively valenced experiences, the scale was designed to assess the subjective intrusiveness of thoughts regardless of their content or valence, capturing the extent to which any thought—negative, neutral, or even positive—is experienced as disruptive or distressing. Nevertheless, the consistently high Cronbach’s alpha values observed across all studies indicate strong internal consistency, affirming a cohesive general construct in addition to the three sub-constructs. Interestingly, Factors 1 and 2 are mainly composed of items originating from the Tinnitus and Metacognitive Questionnaires, reflecting some conceptual overlap with their source measures while supporting the construct validity of the STIQ. Nevertheless, the STIQ, drawing on items adapted from five established instruments, was designed and validated as an integrated tool that consolidates and refines these sources into a concise, transdiagnostic measure of sensitivity to intrusiveness. While the four items within the third factor showed relatively high inter-item correlations, these values were comparable to those observed among other items within the same factor. Nonetheless, future adaptations of the STIQ may consider reducing redundant items to enhance the measure’s efficiency and clarity. This finding shed light not only on the structure of the measure but also on the theoretical construct of sensitivity to intrusiveness. Interestingly, while mind wandering was initially hypothesized to be a significant element in understanding sensitivity to intrusiveness, due to its link with uncontrolled, spontaneous thoughts (93), it was ultimately excluded from the final version of the questionnaire. This exclusion does not imply that mind wandering is necessarily irrelevant to the construct of sensitivity to intrusiveness, but rather it suggests that its inclusion offered limited additional insight when assessed in conjunction with the broader range of STIQ items and dimensions. While there is a correlation between mind wandering and ‘sensitivity to intrusion’, its influence was deemed marginal within the context of the entire questionnaire, leading to its final omission. Future studies should further investigate the role of mind wandering in the experience of intrusiveness, perhaps as a potential fourth factor of the model.

The need for a transdiagnostic assessment tool for intrusive thoughts has been acknowledged in previous studies. Samtani and Moulds (128) conducted a critical review of existing measurement scales used to assess maladaptive repetitive thought across a broad range of clinical disorders. By examining scales used in diverse contexts (e.g., different age groups, rumination, worry) they provided a way to assess repetitive thought in a specific area of interest. McEvoy et al. (112) developed the Repetitive Thinking Questionnaire (RTQ), which is a transdiagnostic tool, not limited to a specific clinical domain. Their research succeeded in distinguishing between repetitive negative thinking and a potentially adaptive variant of repetitive thinking, though it was not experimentally validated across distinct healthy and clinical populations (see also 129). Despite the development of these important transdiagnostic tools, they all focused on the negative manifestations of intrusive thoughts, overlooking the option of neutral or positive repetitive thoughts as an adverse experience. Consequently, their conclusions were confined to negatively laden expressions, such as worry and rumination. Moreover, while these studies examined repetitive negative thinking as a transdiagnostic marker, they failed to effectively showcase its presence within healthy and psychopathological populations. The present study offers the STIQ as a reliable and reliable tool to examine the general intrusive individual experience in various clinical and healthy individuals.

A few potential implications arise from our findings. First, detecting individuals who are more vulnerable to intrusiveness, without being limited to specific psycho-diagnosis, will enhance the therapeutic abilities and early interventions, targeting the specific factors contributing to the sensitivity as the need for control, over-monitoring consciousness, and appraisals of reoccurrence. Additionally, while tools to measure the frequency and content of intrusive thoughts have been previously established, the STIQ is unique in its aim to quantify the extent of intrusiveness experienced, thus capturing individual differences in the sensitivity to such intrusions. Consistent with Samtani et al. (130), who identified a general repetitive negative thinking factor, our findings extend this work by showing that the STIQ captures a broader construct—sensitivity to intrusiveness—that is not limited to negative or repetitive cognitions but encompasses the subjective aversiveness of thoughts of any valence. Future research should focus on exploring the STIQ implementation in other clinical and non-clinical groups, to improve our understanding of the cognitive mechanism and explore the variety in sensitivity to intrusiveness. Future studies should also address potential common-method variance by incorporating complementary methods beyond self-report (e.g., behavioral or experimental measures; 131). In particular, future investigations would do well to include populations with high levels of anxiety, which may provide an opportunity to examine whether the pattern of responses across the STIQ subscales varies significantly by diagnostic category. Within different clinical groups, the STIQ can allow a more accurate perspective on the trait of sensitivity to intrusiveness, potentially allowing more precision therapy. Moreover, it would be beneficial to explore different types of intrusions (neutral, positive, and negative) that might be related to specific disorders, thus, improving our understanding and treatment of those disorders. Additionally, exploring the potential interplay between sensitivity to intrusiveness and other psychological constructs like resilience, coping strategies, or inhibitory control might offer an important research venue.

Alongside the strengths of the present research and the limitations mentioned for the different studies, one general limitation should be noted. Participants of studies 1 and 2 were recruited via PROLIFIC platform, although it is widely regarded as a high-quality online research platform, samples recruited through online panels may not be fully representative of the general population. Importantly, however, the reliability and factor structure of the STIQ were also demonstrated in samples recruited outside of PROLIFIC, including student and clinically diagnosed populations, supporting the robustness of the measure across different recruitment sources.

In conclusion, our study challenges the prevalent assumption that the distressing nature of intrusive thoughts is solely bound to their content. We underscore that while the content may play a role, it is not the exclusive determinant of the distress experienced. Instead, our research introduces and emphasizes the concept of ‘sensitivity to intrusiveness’, a transdiagnostic characteristic that exists independently of specific content. By conceptualizing intrusiveness in this broader context, we offer a more nuanced perspective that accommodates both the negative and non-negative content of intrusions, not specific to a certain type of cognition. The STIQ, introduced in this paper, enables the evaluation and measurement of the intensity with which intrusiveness is felt, positioning it as an individualized variable. This tool has the potential to enhance both research and clinical interventions by identifying individuals who may be vulnerable to the distress caused by intrusive thoughts, even before they develop maladaptive interpretations or coping strategies.

## Data Availability

The original contributions presented in the study are included in the article/**Supplementary Material**. Further inquiries can be directed to the corresponding author.

## Footnotes

1 Note that this does not mean that there was no correlation between mind wondering and other measures of intrusions, but it rather suggests that the contribution of this measure was smaller. See further discussion below.

2 Relationships between the individual STIQ factors and the personality variables are presented in **Table 4**.

3 To our knowledge, only one previous study has suggested that individuals with ADHD (without any comorbid disorders) suffer from intrusive and negative thoughts compared to typically developed individuals (102).

4 Detailed individual-level medication data were not available due to datasharing constraints across collaborating sites.

5 Subscale-level results for Study 4 are presented in **Table 5**.

6 These results were replicated in each of the three subscales of the STIQ except there was no significant difference between HC and ADHD groups (planned comparison number 4) in the Monitoring of thoughts subscale.
