# Supplementary material for: Development and validation of the Sensitivity to Intrusiveness Questionnaire
Source: Front Psychiatry. 2026 Feb 4;17:1599703. doi: 10.3389/fpsyt.2026.1599703 (PMC12914946; doi:10.3389/fpsyt.2026.1599703)
Supplement: Supplementary file 1 [file Supplementaryfile1.docx]

**Supplementary Material 1.** List of initial items loaded into the Sensitivity to Intrusiveness

| **Questionnaire** | **Items** | | | | | |
| --- | --- | --- | --- | --- | --- | --- |
| Wandering Mind Questionnaire | I have difficulty maintaining focus on simple or repetitive work | | | | | |
|  | While reading, I find I haven't been thinking about the text and must therefore read it again | | | | | |
|  | I do things without paying full attention | | | |  |  |
|  | I find myself listening with one ear, thinking about something else at the same time | | | | | |
|  | I mind-wander during lectures or presentations | | | | |  |
|  |  |  |  |  |  |  |
| International Tinnitus Inventory (ITI) | Over the past two weeks, how much have the thoughts affected your sleep? | | | | | |
|  | Over the past two weeks, how much have the thoughts affected your peace of mind? | | | | | |
|  | Overall, how much have the thoughts affected the things you can do? | | | | | |
| Tinnitus Questionnaire | I am aware of the thoughts from the moment I get up to the moment I sleep | | | | | |
|  | I find it harder to relax because of the thoughts | | | | |  |
|  | My thoughts are often so bad that I cannot ignore them | | | | |  |
|  | I am a victim of my thoughts | | |  |  |  |
|  | The thoughts have affected my concentration | | | | |  |
|  | Because of the thoughts I worry that there is something seriously wrong with my mind | | | | | |
|  | On average, to what degree did the thoughts come out of the blue? | | | | | |
|  | Thoughts cause stress | | |  |  |  |
|  |  |  |  |  |  |  |
| OCI-R intrusivness scale | I find it difficult to control my own thoughts. | | | |  |  |
|  | I am upset by unpleasant thoughts that come into my mind against my will. | | | | | |
|  | I frequently get nasty thoughts and have difficulty in getting rid of them. | | | | | |
|  |  |  |  |  |  |  |
| RRS | Think “I won’t be able to do my job if I don’t snap out of this.” | | | | | |
|  | Think about how hard it is to concentrate | | | |  |  |
|  | Think “Why do I always react this way?” | | | |  |  |
|  | Think “I won’t be able to concentrate if I keep feeling this way.” | | | | | |
|  | Think “Why do I have problems other people don’t have?” | | | | | |
|  |  |  |  |  |  |  |
| MCQ-30 | I think a lot about my thoughts | | |  |  |  |
|  | Aware of mind when thinking about problem | | | |  |  |
|  | At fault for bad outcomes related to worry | | | |  |  |
|  | Worrying persists even when trying to stop | | | |  |  |
|  | I cannot ignore my worrying thoughts | | | |  |  |
|  | I monitor my thoughts | | |  |  |  |
|  | I should control my thoughts all of the time | | | |  |  |
|  | I am constantly aware of my thinking | | | |  |  |
|  | I pay close attention to how my mind works | | | |  |  |
|  | Lack of control of thoughts is a weakness | | | |  |  |
|  | I cannot stop worrying | | |  |  |  |
|  | Bad to think certain thoughts | | |  |  |  |
|  | I constantly examine my thoughts | | | |  |  |
|  |  |  |  |  |  |  |
| PSWQ | I find it easy to dismiss worrisome thoughts. | | | | |  |
|  | I notice that I have been worrying about things. | | | | |  |
|  | Once I start worrying, I can't stop. | | | |  |  |

**Supplementary Material 2.** Converted thought-specific items.

| **Original phrasing** |  | **New phrasing** |  |  |
| --- | --- | --- | --- | --- |
| Over the past two weeks, how much has your tinnitus affected your sleep? | | Over the past two weeks, how much have your thoughts affected your sleep? | | |
| Over the past two weeks, how much has your tinnitus affected your peace of mind? | | Over the past two weeks, how much have your thoughts affected your peace of mind? | | |
| Overall, how much has your tinnitus affected the things you can do? | | Overall, how much have your thoughts affected the things you can do? | | |
| Considering everything, how much has your tinnitus changed your enjoyment of life? | | Considering everything, how much have your thoughts changed your enjoyment of life? | | |
| I am aware of the noises from the moment I get up to the moment I sleep | | I am aware of the thoughts from the moment I get up to the moment I sleep | | |
| I find it harder to relax because of the noises | | I find it harder to relax because of the thoughts | | |
| My noises are often so bad that I cannot ignore them | | My thoughts are often so bad that I cannot ignore them | | |
| It takes me longer to get to sleep because of the noises | | It takes me longer to get to sleep because of the thoughts | | |
| I am a victim of my noises | | I am a victim of my thoughts | |  |
| The noises have affected my concentration | | The thoughts have affected my concentration | | |
| Because of the noises, I worry that there is something seriously wrong with my mind | | Because of my thoughts I worry that there is something seriously wrong with my mind | | |
| I notice that I have been worrying about things | | I notice that I have been thinking about things | | |

**Supplementary Material 3.** Rotated components matrix before removing Wandering-mind questionnaire items.

|  | **Factor 1** | **Factor 2** | **Factor 3** | **Factor 4** |
| --- | --- | --- | --- | --- |
| I have difficulty maintaining focus on simple or repetitive work |  | .759 |  |  |
| While reading, I find I haven't been thinking about the text and must therefore read it again |  | .846 |  |  |
| I do things without paying full attention |  | .845 |  |  |
| I find myself listening with one ear, thinking about something else at the same time |  | .864 |  |  |
| I mind-wander during lectures or presentations |  | .830 |  |  |
| Over the past two weeks, how much have the thoughts affected your sleep? | .813 |  |  |  |
| Over the past two weeks, how much have the thoughts affected your peace of mind? | .862 |  |  |  |
| Overall, how much have the thoughts affected the things you can do? | .867 |  |  |  |
| I am aware of the thoughts from the moment I get up to the moment I sleep | .608 |  | .437 |  |
| I find it harder to relax because of the thoughts | .796 |  |  |  |
| My thoughts are often so bad that I cannot ignore them | .597 |  |  |  |
| I am a victim of my thoughts | .475 |  |  | -.413 |
| The thoughts have affected my concentration | .593 |  |  |  |
| Thoughts cause stress | .725 |  |  |  |
| I find it difficult to control my own thoughts. |  |  |  | -.578 |
| I am upset by unpleasant thoughts that come into my mind against my will. | .328 |  |  | -.578 |
| I frequently get nasty thoughts and have difficulty in getting rid of them. |  |  |  | -.705 |
| I think a lot about my thoughts |  |  | .592 |  |
| Aware of mind when thinking about problem |  |  | .630 |  |
| At fault for bad outcomes related to worry |  |  |  | -.632 |
| Worrying persists even when trying to stop |  |  |  | -.811 |
| I cannot ignore my worrying thoughts |  |  |  | -.785 |
| I monitor my thoughts |  |  | .814 |  |
| I am constantly aware of my thinking |  |  | .705 |  |
| I pay close attention to how my mind works |  |  | .852 |  |
| I cannot stop worrying |  |  |  | -.785 |
| I constantly examine my thoughts |  |  | .800 |  |
| Once I start worrying, I can't stop. |  |  |  | -.840 |

***Note.*** The fourth factor contains the 5 mind wandering questionnaire items, which were removed for the next steps of factor analysis (marked in blue color).
